# Indirect versus direct detection methods of *Trichinella* spp. infection in wild boar (*Sus scrofa*)

**DOI:** 10.1186/1756-3305-7-171

**Published:** 2014-04-07

**Authors:** Maria Angeles Gómez-Morales, Alessandra Ludovisi, Marco Amati, Ennio Bandino, Gioia Capelli, Franco Corrias, Luca Gelmini, Alberigo Nardi, Cristina Sacchi, Simona Cherchi, Marco Lalle, Edoardo Pozio

**Affiliations:** 1 Department of Infectious, parasitic and immunomediated Diseases, Istituto Superiore di Sanità, Viale Regina Elena 299, 00161 Rome, Italy; 2 Istituto Zooprofilattico della Sardegna, Nuoro section, Nuoro, Italy; 3 Istituto Zooprofilattico Sperimentale delle Venezie, 35020 Legnaro, Italy; 4 Istituto Zooprofilattico del Lazio e della Toscana, Florence section, Florence, Italy; 5 Istituto Zooprofilattico della Lombardia e dell’Emilia Romagna, Modena section, Modena, Italy; 6 Istituto Zooprofilattico del Lazio e della Toscana, Grosseto section, Grosseto, Italy; 7 Istituto Zooprofilattico della Lombardia e dell’Emilia Romagna, Binago section, Varese, Italy

**Keywords:** *Trichinella* spp, ELISA, Western blot, Wild boar, Meat juice, Chemiluminescence, Prevalence, Surveillance, Italy

## Abstract

**Background:**

*Trichinella* spp. infections in wild boar (*Sus scrofa*), one of the main sources of human trichinellosis, continue to represent a public health problem. The detection of *Trichinella* spp. larvae in muscles of wild boar by digestion can prevent the occurrence of clinical trichinellosis in humans. However, the analytical sensitivity of digestion in the detection process is dependent on the quantity of tested muscle. Consequently, large quantities of muscle have to be digested to warrant surveillance programs, or more sensitive tests need to be employed. The use of indirect detection methods, such as the ELISA to detect *Trichinella* spp. infections in wild boar has limitations due to its low specificity. The aim of the study was to implement serological detection of anti-*Trichinella* spp. antibodies in meat juices from hunted wild boar for the surveillance of *Trichinella* spp. infections.

**Methods:**

Two tests were used, ELISA for the initial screening test, and a specific and sensitive Western blot (Wb) as a confirmatory test. The circulation of anti-*Trichinella* IgG was determined in hunted wild boar muscle juice samples in 9 provinces of 5 Italian regions.

**Results:**

From 1,462 muscle fluid samples, 315 (21.5%, 95% C.I. 19.51-23.73) were tested positive by ELISA. The 315 ELISA-positive muscle fluid samples were further tested by Wb and 32 (10.1%, 95% C.I. 7.29-13.99) of these were positive with a final seroprevalence of 2.2% (95% C.I 1.55-3.07; 32/1,462). *Trichinella britovi* larvae were detected by artificial digestion in muscle tissues of one (0.07%, 95%C.I. 0.01-0.39) out of the 1,462 hunted wild boars. No *Trichinella* spp. larvae were detected in Wb-negative wild boar. From 2006 to 2012, a prevalence of 0.017% was detected by muscle digestion in wild boar hunted in the whole Italian territory.

**Conclusions:**

The combined use of both serological methods had a sensitivity 31.4 times higher than that of the digestion (32/1,462 versus 1/1,462), suggesting their potential use for the surveillance of the *Trichinella* spp. infection in wild boar populations.

## Background

Wild carnivore and omnivore (mainly swine) animals are the main reservoir of nematodes of the genus *Trichinella*, aetiological agents of trichinellosis, a serious and sometimes fatal zoonotic disease [1]. These parasites circulate in all continents with the exception of the Antarctica. In the last 70 years, there has been increasing evidence that the biomass of nematodes of the genus *Trichinella* is greater in wild animals than in domestic animals [2].

Since wild boar (*Sus scrofa*) is frequently consumed by humans, the presence of *Trichinella* spp. in this animal represents a threat for human health [3]. Consequently in the European Union, both bred and hunted wild boar for the market is systematically sampled in slaughterhouses or game-handling establishments to detect *Trichinella* spp. larvae by muscle digestion [4]. However, wild boar for private consumption is exempt from the official controls in some member countries, with the result that these animals bypass veterinary inspection. Consequently*, Trichinella* spp. surveillance programs should be implemented for wild boar in terms of food safety and public awareness increased by informing about the possible risk of acquiring trichinellosis.

For Italian wildlife, the most prevalent species is *Trichinella britovi,* which infect carnivore mammals such as red fox, wolf, and mustelids [5-7]. This parasite species has also been detected in wild boar despite its low prevalence (0.006%-0.017%) [6,8-14].

Serological methods are suitable for surveillance and epidemiological investigations in swine populations [15] and are considered as an appropriate tool for monitoring programs once they are validated by an independent body [4]. ELISA can detect *Trichinella*-specific antibodies in serum and meat juice samples [16], showing a high sensitivity, but may also result in a low specificity due to false-positive reactions especially among swine, which are not reared under controlled conditions [15,17,18].

Therefore, confirmatory testing is required to substantiate the ELISA results [19]. In this regard, several *Trichinella spiralis* proteins recognized by pig sera have been identified by western blotting (Wb) using excretory/secretory antigens (ESA) or a crude worm extract [18-23]. Recently, a distinctive triple-band Wb pattern of *Trichinella* spp. infection has been defined for pigs. However, the visual interpretation of the band pattern is not exempt of problems [18].

The aim of the study was to implement the serological detection of anti-*Trichinella* spp. antibodies in muscle juices from hunted wild boar for the surveillance of *Trichinella* spp. infections. Two tests were used, ELISA for an initial screening test and a specific Wb as confirmatory test. To increase the accuracy of the Wb, a high sensitive revelation system (chemiluminescence) and image software analysis were used; the serological results were then compared with those from the muscle tissue digestion.

## Methods

### Sample collection

**Figure 1:**
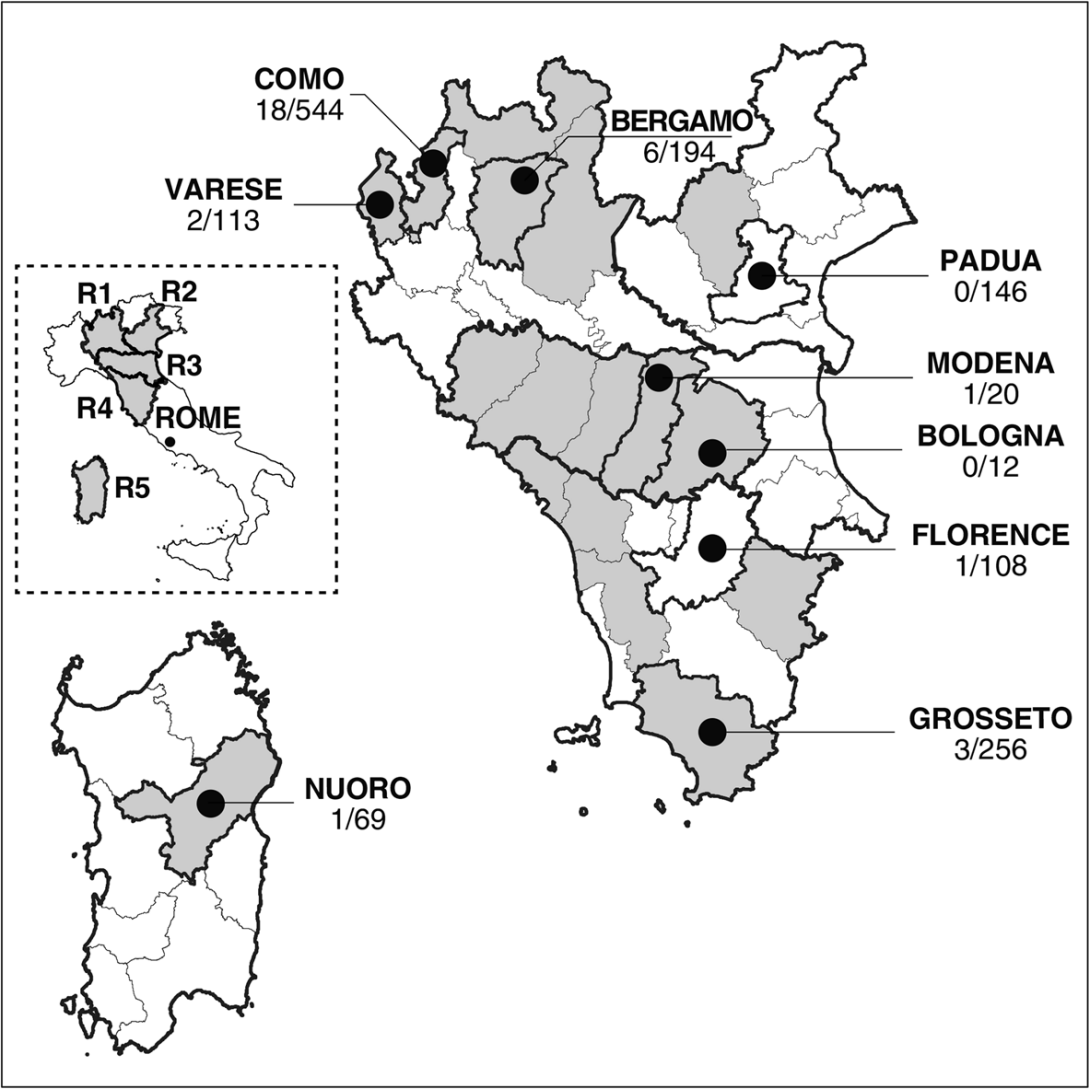
**Western blot positive/tested wild boar (*Sus scrofa*) by Italian provinces.** Black dots, Italian provinces. R1, Lombardy region; R2, Veneto region; R3, Emilia Romagna region; R4, Tuscany region; and R5, Sardinia region. Grey area, indicates the provinces where *Trichinella* spp. have been previously documented in wildlife; white area, the provinces where *Trichinella* spp. have never been documented in wildlife and/or domestic animals in the last 50 years.

During the October 2007 – January 2008 hunting season, diaphragm muscles were collected from wild boar hunted in 9 provinces of 5 regions of northern and central Italy and from Sardinia, selected on the basis of information relating to the presence/absence of *Trichinella* spp. in wildlife [7]: 1) the Alpine area of the Lombardy region (northern Italy) where *T. britovi* has been frequently detected; 2) the Euganean Hills, a group of isolated hills (Veneto region, north-eastern Italy), where *Trichinella* spp. have never been documented, as well as in the whole of the Veneto region after 2005; 3) the Apennine area of the Emilia-Romagna region (northern Italy), where both *T. britovi* and *T. pseudospiralis* have been documented; 4) the Apennine area, province of Florence (Tuscany region, central Italy) where *Trichinella* spp. have never been documented, unlike its bordering provinces where *T. britovi* has been documented; 5) the Grosseto province (Tuscany region), where *T. pseudospiralis* has been recently documented in a wild boar; and 6) the mountain area of the Nuoro province in Sardinia where *T. britovi* has been documented among free-ranging pigs since 2005 to the present [24] (Figure 1).

Diaphragm muscles (50–100 g) were collected from 1,462 wild boars immediately after a hunt, preserved in plastic bags, refrigerated in an ice box during transportation from the field to the laboratory, then again refrigerated at 4°C until digestion, which was performed within 24–48 h after the hunt. Before digestion, muscle was cut into small 5 g pieces and was digested according to the Commission Regulation 2075/2005 [4]. The recovered larvae were counted under a stereomicroscope and the number of larvae per gram (LPG) was determined. The remaining muscle was frozen at −20°C. The frozen muscle was shipped on dry ice from the peripheral laboratories to the Istituto Superiore di Sanità (Rome, Italy). On arrival, the frozen muscle was stored at −20°C until processing. The day before running the serological test, muscle samples were thawed at room temperature and the muscle fluids were collected from the plastic bags and aliquoted at 0.3 ml per vial.

Muscle samples were also collected from 302 pigs from herds kept under controlled management conditions (negative controls). For each animal, 10 g of diaphragm muscle was digested according to a published protocol, as previously mentioned [4]. Muscle fluids from 4 experimentally infected (20,000 larvae) pigs were used as positive controls.

### Trichinella *sp. larva identification*

After digestion, *Trichinella* spp. larvae were washed in a phosphate buffer saline solution, counted in triplicate, and stored in 90% ethyl alcohol for their molecular identification. Single *Trichinella* spp. larvae were identified at the species level by a multiplex PCR analysis, performed according to a validated protocol at the European Union Reference Laboratory for Parasites (Rome, Italy) [25].

### ELISA

The 1/10 diluted muscle fluids from wild boar and control pigs were first tested for the presence of anti-*Trichinella* IgG by ELISA using excretory/secretory antigens (ESA). An in-house ELISA was used in accordance with a previously published validated protocol [17,18]. Since raw optical density (OD) values are absolute measurements that are influenced by ambient temperature, test parameters, and photometric instruments, the results were expressed as a function of the reactivity of the positive control muscle fluid sample with the highest value out of the 4 included in each run of the assay. This control must yield a result that is in the linear range of the measurement [26]. The mean OD values of the control muscle fluids, as well as the mean OD values of the duplicate test muscle fluids, were then calculated, and for each muscle fluid an ELISA index (I_E_) expressed as percentage of positivity was calculated according to the following equation:

$$I_{E}=\frac{\mathrm{mean}\;\mathrm{OD}\;\mathrm{value}\;\mathrm{of}\;\mathrm{duplicate}\;\mathrm{sample}‒\mathrm{OD}\;\mathrm{blank}}{\mathrm{mean}\;\mathrm{OD}\;\mathrm{value}\;\mathrm{of}\;\mathrm{the}\;\mathrm{highest}\;\mathrm{positive}\;\mathrm{control}‒\mathrm{OD}\;\mathrm{blank}}\times 100$$

The cut-off value for OD, calculated as the mean (± 3 SD) of the OD values of the 302 muscle fluids from *Trichinella*-negative pigs was 24.8%.

### Western blot

All ELISA-positive muscle fluids, were then tested by Wb according to a previously published validated protocol [18], but using a high sensitive revelation system based on chemiluminescence, as the level of antibodies in muscle fluids is lower than that in sera [16]. Furthermore, to assess the quality of the electrophoretic transfer of gels, a pre-stained standard of molecular weight was used (Precision Plus Protein™ WesternC™ Standars, Bio-Rad, Hercules, CA, USA) in each run. An experiment was considered to be valid when all of the pre-stained protein standards (250, 150, 100, 75, 50, 37, 25 and 20 kD) were separated and transferred onto the nitrocellulose membrane, and the relative mobility of each standard was within the standard range previously established by 3 independent experiments. The nitrocellulose filters were blocked with 5% skimmed milk in 1 X Tris-Borate saline-Tween (TBST, 50 mM Tris pH 8.0, 150 m NaCL, 1% Tween 20) at 4°C overnight and washed 3 times with 1 X TBST. Each nitrocellulose filter was cut into strips, each of which was then incubated with 1/10 muscle fluid with 3% (w/v) skimmed milk (Sigma-Aldrich, Saint Louis, MO, USA) in 1 X TBST at RT for 1 h. After washing 3 times with 1 X TBST, the pre-stained protein standard strip was incubated with Precision Protein™ Strep Tactin-HRP conjugated at 1:10,000 dilution, for 1 h. The other strips (previously incubated with the muscle fluid) were incubated with a 1:5,000 dilution of goat anti-swine IgG conjugated with horseradish peroxidase (Bio-Rad, Hercules, CA, USA) for 1 h. To reveal proteins with high efficiency, the LiteAblot® Plus chemiluminescence system (Euroclone, Pero, Milan, Italy) was added to the strips for 5 min. The proteins were then visualized on a ChemiDoc™ XRS System (Bio-Rad) and images were analyzed using the Image Lab™ software version 4.0 (Bio-Rad). The experiment was considered valid when the relative mobility (Rf) of the proteins which define the pattern of *Trichinella* spp. infections [18], were within the range previously established by 3 independent experiments for each positive control (first band from 0.410 to 0.524 mm; second band from 0.365 to 0.499 mm; and third band from 0.319 to 0.428 mm). The positivity/negativity of each wild boar muscle fluid was then determined by comparing the Rf value of each sample with the positive control on the same blot, and the corresponding MW calculated by the Image Lab™ software version 4.0 (Bio-Rad).

### Statistical analysis

Two-tailed Student’s t-tests were performed using GraphPad Prism (Version 3, Graph-Pad Software, San Diego, CA, USA) to compare the test performance. The differences of prevalence obtained by serology (Wb) and digestion method were tested using the chi-square test.

### Ethical statement

The wild boar meat sampling was performed according to the Commission Regulation 2075/2005 [4].

## Results

### Artificial digestion

*Trichinella* spp. larvae were detected in one (0.07%, 95% C.I. 0.01-0.39) out of the 1,462 muscle samples from hunted wild boar. The *Trichinella* sp. positive wild boar with a larval burden of 0.15 LPG was hunted in the Varese province (Figure 1). No *Trichinella* sp. larva was detected in the 302 pig muscle samples from herds kept under controlled management conditions. The *Trichinella* sp. larvae isolated from the wild boar were identified as *T. britovi* (data not shown).

### ELISA

**Figure 2:**
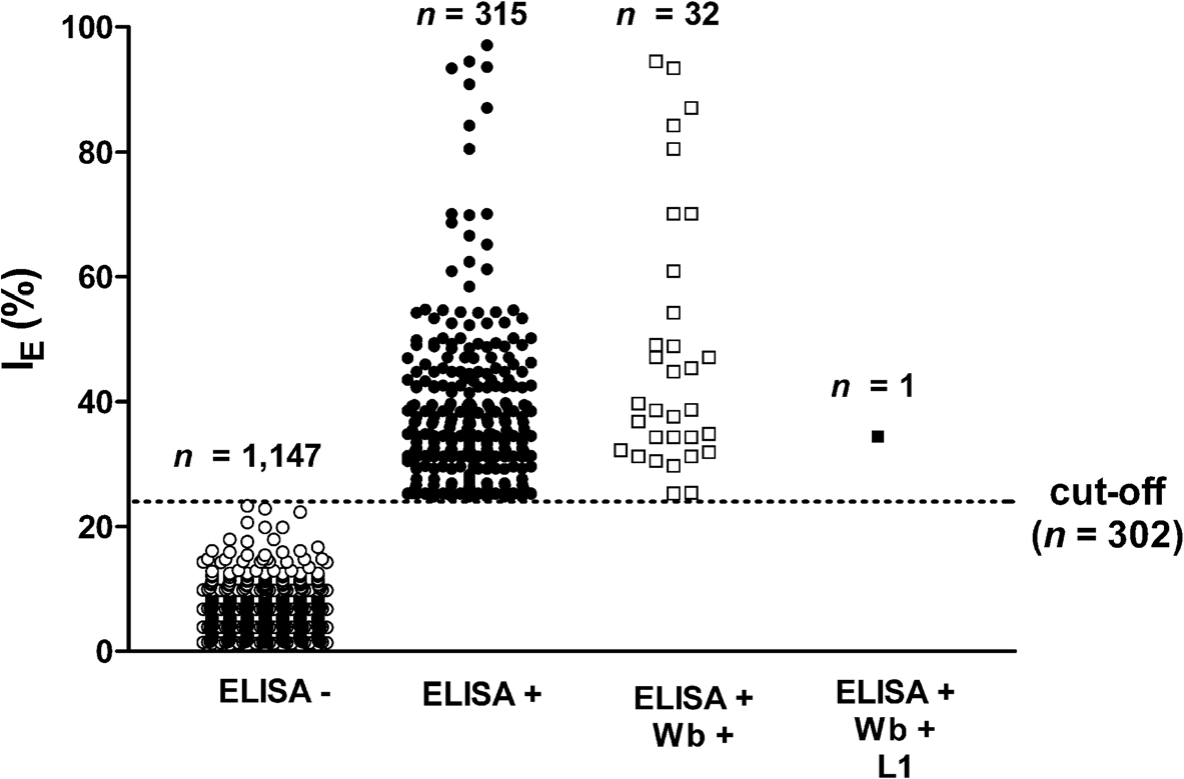
**Scatter plot of the ELISA index (I**_**E**_**) for anti-*Trichinella *spp. IgG in wild boar.** ELISA-, muscle fluids resulted negative by ELISA; ELISA+, muscle fluids resulted positive by ELISA; Wb+, muscle fluids resulted positive by ELISA and Western blot; L1, wild boar tested positive for *Trichinella britovi* larvae by artificial digestion.

As shown in Table 1 and Figure 2, out of the 1,462 muscle fluids from wild boar, 315 (21.5%, 95% C.I. 19.51-23.73) tested positive by ELISA (173, 54.9%, with an I_E_ from 24.8 to 34.8; 84, 26.8%, with an I_E_ from 34.8 to 44.8; 40, 12.7%, with an I_E_ from 44.8 to 54.8; and 18, 5.7%, with an I_E_ > 54.8). The wild boar which tested positive for *T. britovi* larvae showed an I_E_ of 34.0. The prevalence of ELISA-positive muscle fluids ranged from 7.0% to 33.5% with respect to the province of origin (Table 1). None of the muscle fluids from the 302 control pigs tested positive by ELISA.

**Table 1:** **Prevalence of anti-*Trichinella *** **IgG detected in muscle fluids of wild boar hunted in Italy**

**Region of origin province**	**ELISA positive/tested wild boar (%)**	**Western blot positive/ELISA positive wild boar (%)**	**Western blot positive/tested wild boar (%)**	**Previous reports of *Trichinella *** **spp. in wildlife**
Lombardy				
Bergamo	65/194 (33.5)	6/65 (9.2)	6/194 (3.1)	Yes
Como	159/544 (29.2)	18/159 (11.3)	18/544 (3.3)	Yes
Varese	31^a^/113 (27.4)	2^a^/31 (12.5)	2^a^/113 (1.8)	Yes
Veneto				
Padua	21/146 (14.4)	0.0/21	0.0/146	No^b^
Emilia Romagna				
Bologna	1/12 (8.3)	0.0/1	0.0/12	Yes
Modena	4/20 (20.0)	1/4	1/20 (5.0)	Yes
Tuscany				
Florence	12/108 (11.1)	1/12 (8.3)	1/108 (0.9)	No^c^
Grosseto	18/256 (7.0)	3/18 (16.7)	3/256 (1.2)	Yes
Sardinia				
Nuoro	5/69 (7.2)	1/5	1/69 (1.4)	No^d^
Total	315^a^/1,462 (21.5)	32^a^/315 (10.1)	32^a^/1,462 (2.2)	

^a^*Trichinella britovi* larvae detected in muscle tissues of a single animal.

^b^none reported in the Padua province and Veneto region since 2005 (see Figure 3).

^c^none reported in the Florence province, but *T. britovi* has been documented in the neighbouring provinces (see Figure 3).

^d^none reported in wild animals before 2011, but *T. britovi* has been documented in free-ranging pigs from 2005 to the present.

### Western blot

**Figure 3:**
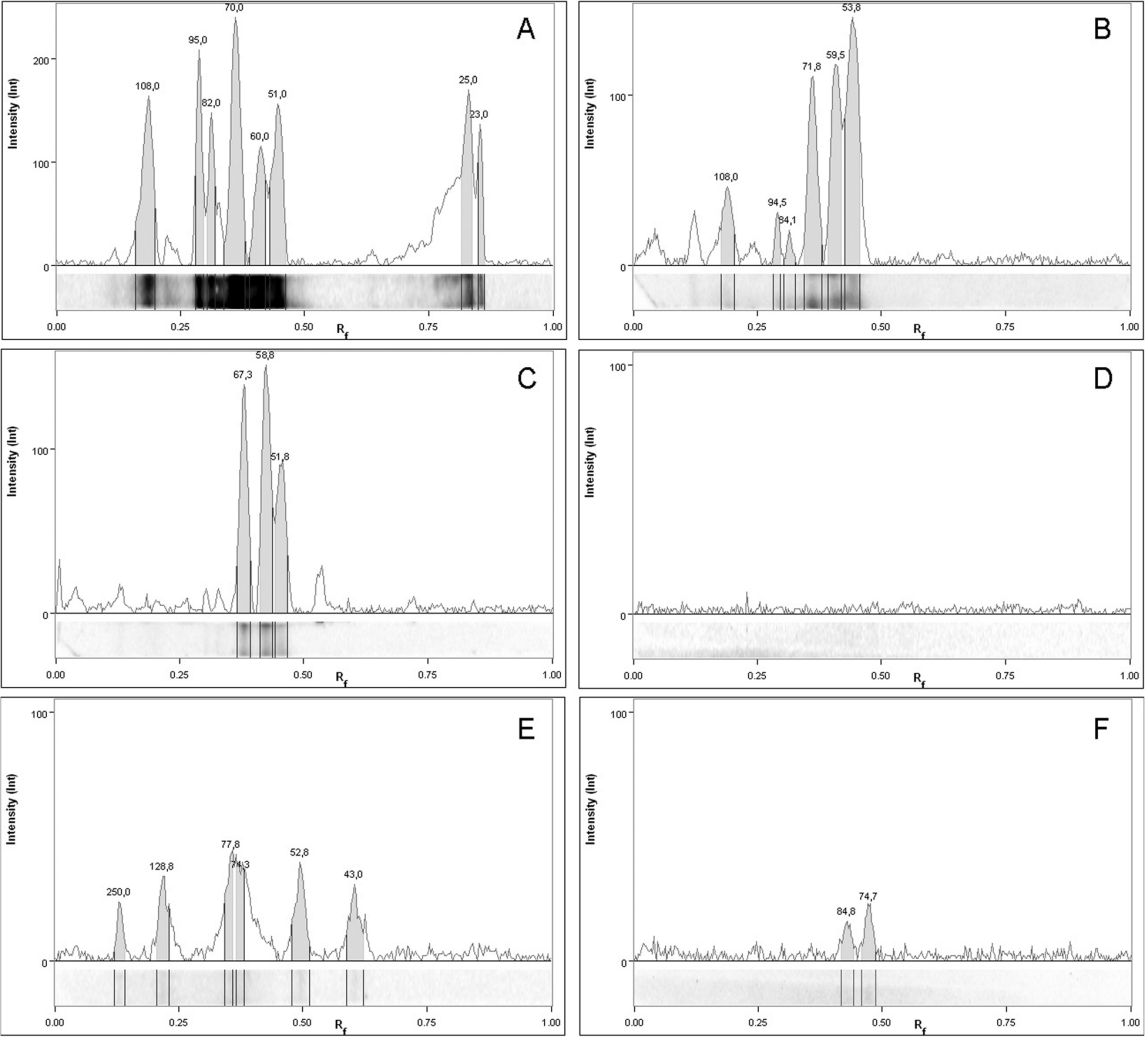
***Trichinella spiralis *excretory/secretory antigens recognized by Western blot in muscle fluids from wild boar.** Signal intensities and relative migration values (Rf) of the proteins recognized by: a positive control muscle fluid from an experimentally infected pig, panel **A;** the muscle fluid from the wild boar with 0.15 LPG in the diaphragm, panel **B;** a low positive muscle fluid from a wild boar, panel **C;** a muscle fluid from an uninfected control pig, panel **D;** and the false positive muscle fluids from two ELISA-positive wild boars, panels **E** and **F**. Numbers above each peak refer to the software deduced molecular weights of the bands.

The 315 ELISA-positive muscle fluids were further tested by Wb and 32 (10.1%, 95% C.I. 7.29-13.99) including the wild boar which had been tested positive by digestion, showed the 3 band pattern which is considered to be diagnostic for the presence of anti-*Trichinella* IgG (Table 1; Figures 2 and 3). No positive muscle fluid was detected by Wb from wild boar hunted in the Euganean area. In the Alpine areas of the Lombardy region, a prevalence from 1.8% to 3.3% of anti-*Trichinella* IgG was detected by Wb in the wild boar population according to the province of origin (Figure 1). A prevalence of 5.0% was detected by Wb in wild boar hunted in the Apennine area of the Emilia Romagna region (Figure 1). A prevalence of 0.9% and of 1.2% was detected by Wb in wild boar hunted in the Florence and Grosseto provinces, respectively (Figure 1). The muscle fluid of 1 (1.4%) out of 69 wild boars from Sardinia was positive for anti-*Trichinella* IgG by Wb (Figure 1).

## Discussion

The Wb results show a total prevalence of anti-*Trichinella* IgG in the tested wild boar populations of Italy of 2.2% (95% C.I. 1.55-3.07; 32/1,462), which is 31.4 times higher than the prevalence detected by digestion of muscle tissues (0.07%; *P* < 0.001). The serological prevalence varies according to the hunting areas and is consistent with the information on the circulation of these zoonotic parasites in wildlife (Figure 1) [5-7].

The detection of only a single positive wild boar (0.07%) by the digestion test cannot be attributed to poor test performance, since all the laboratories performing the test are accredited according to ISO 17025. Additionally, a large quantity of preferential muscles from each animal was tested in two different laboratories, and a worm burden of 0.15 LPG was detected suggesting a good sensitivity.

The different prevalence obtained by digestion and serology (0.07% versus 2.2%, *P* < 0.001) can be explained by the analytical sensitivity of the artificial digestion test which is of 1 LPG [26-28], whereas, the analytical sensitivity of the ELISA has been reported to be 0.01 LPG [26,27]. No data exist on the analytical sensitivity of Wb, namely the minimum detectable antibody level by Wb and the number of LPG in preferential muscles. However, the study results show that anti-*Trichinella* IgG can be detected by Wb in the muscle juice of a wild boar with 0.15 LPG suggesting a good sensitivity for this test when chemiluminescence was used. Moreover, visual observation does not permit the Wb band pattern to be objectively evaluated. To achieve a high accuracy in the interpretation of the Wb pattern, it is necessary to carefully measure the relative migration distances of the proteins to infer the molecular weight. Consequently, the interpretation of the Wb pattern is time consuming and needs experienced personnel. These problems can be overcome by the use in reference laboratories of an image analyzing software, which could permit the standardization of the procedure and also an accurate interpretation of the results. The serological specificity has been increased by coupling the ELISA with the Wb, and the Wb sensitivity has been increased by using a high sensitive revelation system based on chemiluminescence (Figure 3).

The discordance between the prevalence obtained by serology and digestion (2.2% versus 0.07%) could be related also to biological and immunological reasons. Experimental infections show a low infection burden and a short survival time of *T. britovi* larvae in swine muscles. At two months post infection the number of *T. britovi* larvae in swine muscles ranges from 1.6 to 280 times lower than that of *T. spiralis*[16]. Furthermore, re-infections cannot be excluded. A primary infection with *T. spiralis* in pigs induces nearly absolute resistance to the muscle establishment of larvae from new infectious worms, and the fecundity of female worms recovered from immune pigs was reduced by 75% in comparison to controls [29]. Despite the low infectivity of *T. nativa* for swine, primary inoculation with this species resulted in high levels of immunity against challenge infection with *T. spiralis*; this immunity was expressed in accelerated expulsion of challenge adults from the intestine and reduced numbers of muscle larvae [30].

The study results show that anti-*Trichinella* IgG can be easily detected in the muscle juice. Furthermore in wild animals, it is easier to collect muscle juices of good quality than to collect serum samples of good quality, namely not haemolysed and without bacteria. Once this indirect detection system (ELISA + Wb) is validated in other areas with different degrees of prevalence, it could be included as a tool for the surveillance of *Trichinella* spp. infection in wild boar and in domestic pig populations at risk for these zoonotic parasites, namely backyard and free-ranging pigs [31].

Recently, a *Trichinella* seroprevalence of 3.5% (95% C.I. 0.0–8.0) was detected in sera from wild boar hunted in the Australian mainland, by an ELISA coupled with a Wb in a similar way to that used in the present study. The seroprevalence was significantly higher (P < 0.05) than that of the artificial digestion (0.0%, 95% C.I. 0.0–1.1) [32]. In a similar way to the results of the present study, *Trichinella papuae* larvae were detected in a muscle sample from only a single wild boar hunted in the Gabba Island in the Torres Strait not far from the Australia mainland. Here only serologically positive animals have been detected.

In another survey carried out on wild boar of south-central Spain [33], a different prevalence was observed between serology and digestion, than in the present study: i) Wb has been performed on only a percentage of ELISA-positive sera; ii) no chemiluminescence has been used to increase the Wb sensitivity; and iii) both *T. britovi* and *T. spiralis* is known to circulate in the investigated region, the latter being more infectious for swine. In North Carolina, a serological prevalence of 13.3% was detected in feral pigs by a commercial ELISA kit, but ELISA-positive samples were not confirmed by Wb and no information is available on *Trichinella* spp. larvae in muscles [34].

*Trichinella britovi* was the only species detected in Italian wildlife up to few years ago when *T. pseudospiralis* began to be documented in northern and central Italy [7,35]. The prevalence of *Trichinella* spp. infections in the wild boar populations of Italy has been always extremely low. From 1985 to 1999, *T. britovi* was detected in 9 (0.002%) out of 370,000 hunted wild boars [6]. In the last 13 years, there has been an increase of the wild boar population in Italy and, in parallel, an increase of the number of hunted wild boar and an increase of wild boar tested for *Trichinella* spp. per year according to the Commission Regulation 2075/2005 [4]. From 2007 to 2012, a total prevalence of 0.017% (95% CI 0.0001-0.0002; 47 positive/268,200 tested), was detected [8-14], which is significantly higher (*P* < 0.001) than the prevalence detected in the previous period, despite the great differences among the Italian regions (data not shown).

## Conclusions

The highly specific and sensitive indirect detection system (ELISA + Wb) used in the present work, represents a very useful tool to increase the efficacy of surveillance programs. In fact, this detection system may allow the identification of the areas where *Trichinella* spp. are already circulating but where no larva have yet been detected by direct methods.

## Competing interests

The authors declare that they have no competing interests.

## Authors’ contributions

All the authors have contributed significantly to this study. MAGM and EP designed the study, performed the data analysis and interpretation, and prepared the manuscript. AL, MA, SC and ML performed laboratory tests and processed the data. EN, GC, FC, LG, AN and CS organized the sample collection on the field and the muscle digestion in the peripheral laboratories. All authors read and approved the final manuscript.
